# Towards Effective Photothermal/Photodynamic Treatment Using Plasmonic Gold Nanoparticles

**DOI:** 10.3390/ijms17081295

**Published:** 2016-08-09

**Authors:** Alla Bucharskaya, Galina Maslyakova, Georgy Terentyuk, Alexander Yakunin, Yuri Avetisyan, Olga Bibikova, Elena Tuchina, Boris Khlebtsov, Nikolai Khlebtsov, Valery Tuchin

**Affiliations:** 1 Research Institute for Fundamental and Clinical Uronephrology, Saratov State Medical University, n.a. V.I. Razumovsky, 410012 Saratov, Russia; gmaslyakova@yandex.ru (G.M.); vetklinikanew@mail.ru (G.T.); 2 Research-Education Institute of Optics and Biophotonics, Saratov National Research State University, 410012 Saratov, Russia; olyabibikova@gmail.com (O.B.); tuchinvv@mail.ru (V.T.); 3 Institute of Precision Mechanics and Control, RAS, 410028 Saratov, Russia; anyakunin@mail.ru (A.Y.); yuaavetisyan@mail.ru (Y.A.); 4 Artphotonics GmbH, 12489 Berlin, Germany; 5 Optoelectronics and Measurement Techniques Laboratory, University of Oulu, 90014 Oulu, Finland; 6 Institute of Analytical and Bioanalytical Chemistry, Ulm University, 89081 Ulm, Germany; 7 Department of Biology, Saratov National Research State University, 410012 Saratov, Russia; firstflower@yandex.ru; 8 Institute of Biochemistry and Physiology of Plants and Microorganisms, RAS, 410049 Saratov, Russia; khlebtsov_b@ibppm.ru (B.K.); khlebtsov@ibppm.ru (N.K.); 9 Department of Nano- and Biomedical Technologies, Saratov National Research State University, 410012 Saratov, Russia; 10 Interdisciplinary Laboratory of Biophotonics, National Research Tomsk State University, 634050 Tomsk, Russia

**Keywords:** Au nanoparticles, nanorods, nanostars, silica, nanocomposites, photothermal therapy (PPT), photodynamic therapy (PDT), pathogens, tumors, cell optoporation

## Abstract

Gold nanoparticles (AuNPs) of different size and shape are widely used as photosensitizers for cancer diagnostics and plasmonic photothermal (PPT)/photodynamic (PDT) therapy, as nanocarriers for drug delivery and laser-mediated pathogen killing, even the underlying mechanisms of treatment effects remain poorly understood. There is a need in analyzing and improving the ways to increase accumulation of AuNP in tumors and other crucial steps in interaction of AuNPs with laser light and tissues. In this review, we summarize our recent theoretical, experimental, and pre-clinical results on light activated interaction of AuNPs with tissues and cells. Specifically, we discuss a combined PPT/PDT treatment of tumors and killing of pathogen bacteria with gold-based nanocomposites and atomic clusters, cell optoporation, and theoretical simulations of nanoparticle-mediated laser heating of tissues and cells.

## 1. Introduction

Currently, gold nanoparticles (AuNPs) of different size and shape are often used as photosensitizers for cancer plasmonic photothermal (PPT) therapy [1,2], for cell optoporation [3,4] and pathogen killing [5]. It is mandatory to analyze the underlying mechanisms to increase the selectivity of AuNP accumulation in tumors, to enhance treatment efficiency and photo-cytotoxicity, as well as to study other critical effects of laser radiation and AuNPs interaction with tissues and cells that are of great importance for laser treatments.

Progress in the application of plasmonic AuNPs for laser hyperthermia of tumor tissues is due to the multiplicative effects of increased local absorption of laser radiation by plasmonic AuNPs [6] and their targeted delivery [7]. The combined impact of these factors has revolutionized the traditional and widespread laser hyperthermia of tissues. To take advantage of these mechanisms, it is necessary to develop multi-scale models and methods for calculation of the temperature fields on macro- and micro-scales. Furthermore, the models should be adopted for practical application to large size objects at the second- and minute-time scale, whereas the nano-, pico- and femtosecond laser pulses are applied to individual particles. Recently, significant advances were achieved in the development of mathematical modeling methods of photothermal effects of nanoparticles (NPs) in tissues and cells [8,9]. It was shown that local inhomogeneities of the temperature fields lead to new laws of the distribution and dynamics of the Arrhenius damage integral [10,11], which provide a more appropriate selection of nanoparticle dimension and light exposure to gain precise control of hyperthermia with minimal energy consumption.

Laser induced cell optoporation by local heating of AuNPs was proven to be a promising approach to deliver exogenous molecules into cells. Theoretical estimations were used to build a strategy to increase laser cell membrane optoporation efficiency that strongly depends on the laser beam intensity and AuNP optical and thermal properties [12,13]. To achieve the optimal AuNP–cell interaction, sphere-, rod- and star-shaped AuNPs with the different plasmon-resonant peaks were fabricated and functionalized with different ligands [14,15].

One of the potential applications of AuNPs is to kill pathogens, as well as to regulate the number of opportunistic microorganisms. Many differently structured AuNPs have been studied to potentiate antimicrobial PDT by improving photosensitizer solubility, photochemistry, photophysics and targeting [16,17]. Potential ways to improve method are to increase local concentration of the photosensitizer via targeted delivery of nanoparticles, to provide selective interaction with the cell wall of bacteria, and the resonance NP heating under laser radiation.

Currently, the practice of cancer plasmonic photothermal (PTT) and photodynamic (PDT) therapies has advanced in leaps and bounds due to the wide application of multifunctional plasmonic nanocomposites (NCs) and fluorescent photodynamic molecules. Unfortunately, a porphyrin-based PDT can be practical only for tumors on or under the skin or mucosa of the oral or internal organs, as it absorbs light wavelengths of less than 640 nm [18]. The usage of gold NCs for effective laser heating and photodynamic impact on tumor tissue was demonstrated recently [19,20]. Nevertheless, further studies are needed to improve the therapeutic protocols by adjusting light delivery, its power density, and irradiation doses.

In this review, we summarize our recent theoretical, experimental, and preclinical results on light activated interaction of AuNPs with tissues and cells. Specifically, we discuss a combined PPT/PDT treatment of tumors and killing of pathogenic bacteria with gold-based nanocomposites and atomic clusters, cell optoporation, and theoretical simulations of nanoparticle-mediated laser heating of tissues and cells.

## 2. Nanocomposites Based on Au Nanoparticles and Nanoclusters

The PTT is a promising strategy to destroy selectively tumor tissue [21]. This form of therapy is achieved by the conversion of light to thermal energy to heat up tissues by using light-absorbing materials. In order to maximize the photothermal response of the materials to laser irradiation, it is essential to maximize their absorption in the near-infrared (NIR) region [22]. Gold nanostructures are good candidates for photothermal cancer treatment, due to their high biocompatibility, ease of surface functionality and tunable localized surface plasmon resonance (LSPR) absorption band. Various geometries of gold nanoparticles have been previously synthesized, including nanorods, nanoshells and nanocages, in order to use them for PTT in the NIR region [23,24,25], where tissue light scattering and absorption are minimal. However, this therapy may still be challenged for the tumors located deep in the tissues. Many papers have been focused on destruction of deep-located tumors using optical fibers. Recently, Bhatia et al. suggest implanting an 808 nm-laser source for effective intratissue PTT using Au nanorods (AuNRs) as a photothermal agent [26]. Despite recent progress in this field and successful experiments in vitro and in vivo using tumor-breeding mice models [27], it has now become obvious that using PTT alone is an ineffective way to totally eliminate large, solid tumors and additional treatment is usually needed to stop tumor progression. For such a purpose, Hauck et al. [28] used AuNRs combined with chemotherapeutic drug cisplatin. In this study, the cell culture was incubated with AuNRs and cisplatin was added separately before laser irradiation. The cancer cells’ viability after this treatment was 78% less than that after chemotherapy alone and 84% less than after PTT alone. This fact confirms the synergistic effect of combined PPT and chemotherapy.

In the past few years, hybrid Au nanoparticle systems have attracted significant interest, as they combine the outstanding optical properties of nanoparticles with dyes or drugs [29]. By use of smart bioconjugation techniques [30], Au nanoparticles can be functionalized with a set of different molecules, enabling them to perform targeting, diagnostic, and therapeutic functions in a single treatment procedure. This class of multifunctionalized nanoparticles has found exciting applications in proof-of-concept theranostic experiments [31]. For example, Tingting Wang et al. [32] have demonstrated DOX conjugated PaaPEG-AuNRs for combined cancer PTT and drug delivery. Jianliang Shen et al. [33] suggested novel dual-modality nanoparticles with the capability of gene silencing through the incorporation of siRNA and AuNRs mediated PTT. In this paper, the effective suppression of mRNA and corresponding protein expression was due to intracellular delivery of a siRNA against PKM2. The combined activity of PKM2 inhibition and plasmonic heating dramatically reduced the viability of breast cancer cells. In paper [34], the Al(III) phthalocyanine chloride tetrasulfonic acid functionalized AuNRs were applied for fluorescent imaging and PTT in vivo for mouse model. Three-modality nanocomplex based on AuNRs and protoporphyrin IX was recently applied for in vivo SERS detection, fluorescence imaging, and PDT [35]. Shouju Wang et al. reported on single NIR laser induced PDT/PTT therapy using chlorin e6 functionalized gold nanostars [36]. The problem of dye quenching near the metal surface together with low adsorption capacity of colloidal particles are strong limitation factors towards synthesis of effective complexes of metallic nanoparticles with PDT dyes. Recently, we suggested [37,38] composite nanoparticles consist of AuNRs that are coated with a mesoporous silica shell functionalized with a photosensitizer. This approach enables one to overcome the limitations concerning dye quenching and the low loading capacity of the nanocomposites. By using a NIR absorbing Au core and a mesoporous silica shell doped by hematoporphirin (HP) molecules, one enables the preparation of multifunctional nanoparticles for combined PTT/PDT therapy and enhanced photodynamic efficiency.

**Figure 1:**
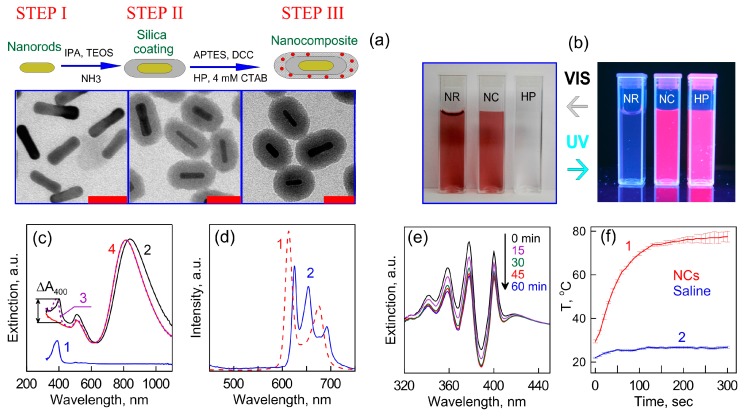
Schematic representation of the basic steps in fabrication of nanocomposites AuNR/SiO_2_-HP containing a plasmonic core, a primary silica shell, and a secondary mesoporous silica shell doped with HP molecules. Scale bars are 50 nm (**a**); Photos of cuvettes with silica-coated AuNRs (NR), nanocomposites (NCs), and an HP solution taken under visible (VIS) and UV light illuminations (**b**); Extinction spectra of HP (curve 1), NC (curve 2), and NR (curve 4) solutions; the spectrum 3 (dotted curve 3) was obtained by the superposition of spectra 1 and 4. The difference ∆*A*_400_ is roughly equal to the extinction maximum of HP with a concentration of 7 mg/L (curve 1) (**c**); Fluorescence spectra of HP (1) and NC (2) solutions measured under 405-nm excitation (**d**); For these measurements, all solutions were diluted 1:16. Absorption spectra of a mixture of ABDA and NCs under illumination by a 633-nm laser for different exposures from 0 to 60 min (**e**); Time-dependent increase in the temperature of NC suspension (1) and saline (2) under NIR laser (808 nm, 2 W/cm^2^) irradiation (**f**). Reproduced from [37,38] with permission from the Springer and Wiley.

The three basic steps of nanocomposite (NC) synthesis together with TEM images of resultant particles are presented in Figure 1a. Step 1 is the fabrication of Au nanorods with plasmon resonance in NIR spectral region. In Step 2, the AuNRs were coated with the primary SiO_2_ shell, which serves as a spacer between the Au nanorods and dye molecules. Finally, an additional layer of a mesoporous silica with HP molecules embedded inside was formed. As a result, we obtain AuNR/SiO_2_-HP composite particles consisting of the plasmonic AuNR core, the first HP-free silica layer, and the second HP-loaded mesoporous silica layer. Figure 1b shows photographs of cuvettes with silica coated nanorods, nanocomposites and HP solution under visible and UV illumination. The NR and NC samples have a red-brown color due to characteristic transversal mode in extinction spectra of AuNRs located at 510 nm while the HP solution looks water-white. The extinction spectra of the nanoparticle solution (Figure 1c) had an intense longitudinal resonance in the region 810–820 nm. In addition, as distinct from the AuNRs spectra, the NC spectrum has a characteristic peak at 400 nm related to absorption of HP molecules inside a nanocopmosite. Taking into account molecular absorption coefficient, we can assume the concentration of included HP molecules to be 7 mg/mL or 3.8 × 10^3^ HP molecules per particle. The visual inspection of the cuvettes with samples (Figure 1b) under UV light showed red fluorescence of nanocomposites and the HP solution, whereas the silica coated Au nanorod suspension looks colorless. To indicate the difference in the fluorescence for HP molecules in solution and in the NC, the emission spectra of diluted solutions were measured. Figure 1c shows spectra taken during excitation with 405 nm-light. We observed the difference in both spectral shapes and intensities for the HP solution and the NC. For example, fluorescence spectra for HP solution has two peaks at 615 and 675 nm while the NC fluorescence spectrum exhibits three peaks located at 630, 650 and 690 nm. On the other hand, the intensity integral over all wavelengths is approximately the same for both cases.

The photo-oxidation of the 9,10-anthracenediyl-bis (methylene) dimalonic acid (ABDA) during the illumination of samples with 625 nm light was measured to detect the singlet oxygen generation. The results of measurements for singlet oxygen generation are shown in Figure 1d. It is clearly seen that the characteristic absorbance peaks of ABDA gradually decrease with an increase of irradiation time. Since the silica shell serves as a protection layer between the metal surface and dye molecules, we did not observe a significant difference between the photodynamic activities of free HP and nanocomposite solutions.

To compare photothermal conversation mediated by nanocomposites and plasmonic core (AuNRs), we measured the in-depth temperature distributions and the time dependent temperature changes in nanoparticle solutions under NIR light irradiation. During plasmonic heating of nanocomposites, the maximal temperature was about of 72 °C (Figure 1d). In the control test of tubes with saline, the temperature only reached 25 °C during 300 s of irradiation with a laser power density of 2 W/cm^2^.

On a whole, we can conclude that our synthetic procedures allow us to obtain nanostructures possessing three important optical modalities—fluorescence under UV irradiation, generation of singlet oxygen under 625 nm irradiation and NIR mediated photothermal conversion.

Au nanoclusters are a new type of luminescent nanomaterials, usually comprising Au nanoparticles smaller than 2 nm, and are typically composed of a few to about 100 gold atoms. The AuNCs are distinguished from the other nanomaterials by their strong photoluminescence, large Stokes shift and high emission rates [22]. The luminescent gold nanoclusters provide the bridge between atomic and nanoparticles behavior in noble metals and exhibit molecule-like photophysical properties, large surface-to-volume ratios, easy fictionalization and color tunability [39]. In contrast to many publications on multifunctional composites based on plasmonic nanoparticles [40], analogous nanocluster-based multifunctional theranostic nanocomposites have been developed in a few reports only. Perhaps, the first report on theranostic application of Au nanoclusters was published by Haiyan Chen et al. [41]. In this paper, Au-BSA-FA nanoclusters were loaded by a chemotherapeutic drug doxorubicin and a near infrared fluorescent dye MPA. The Au-BSA-FA-MPA and Au-BSA-FA-DOX were successfully applied for in vitro and in vivo tumor diagnostics and therapy with cancer cells and a xenografted mice model. Ding and Tian [42] applied Au-BSA-FITC-FA nanoclusters to specific bioimaging and biosensing of cancer cells, where Au nanoclusters produced a reference fluorescent signal, FITC allowed for pH monitoring, and FA acted as targeting molecules. In the Ref. [43], unique nucleus-targeting gold nanoclusters were made and applied for in vitro and in vivo fluorescence imaging, RNA delivery, and PDT of cancer cells. The main advantage of the TAT peptide-Au nanoclusters is their high accumulation rate into the cytoplasm region and a significant accumulation into the nucleus. Finally, Cui and co-workers [44] developed multifunctional NCs comprising GSH-cupped Au nanoclusters that further were coupled with FA and PEG followed by embedding photosensitizer chlorin e6 into PEG shell. The obtained Au-GSH-FA-PEG-Ce6 complexes were successfully applied to simultaneous in vitro and in vivo imaging and photodynamic therapy of cancer cells and tumors in mice.

## 3. Multiscale Mathematical Modeling of Temperature Field of Tissues and Cells Doped by Plasmonic Nanoparticles

**Figure 2:**
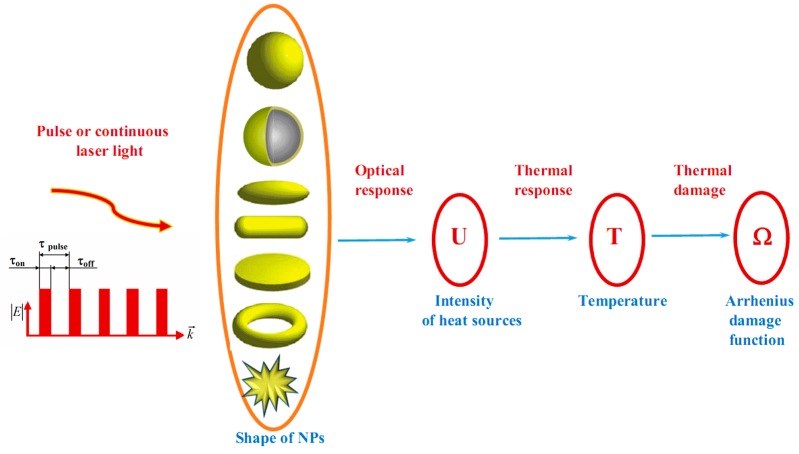
The general scheme of mathematical modeling of nanoparticle mediated laser photothermal treatment of cells or tissues. Here U = ε″ω|*E*|^2^/8π is the local value of intensity of heat sources caused by the absorption of laser radiation(see, e.g., [55]); ε″ is the imaginary part of dielectric constant of NP, ω and *E* are the angular frequency and the local value of electric field amplitude diffracted into NP, respectively.

Recently, there has been significant progress in the development of theoretical analysis methods for examining the photothermal effects of nanoparticles (NPs) on tissues and cells [45,46,47,48,49,50,51,52,53,54,55,56]. The general scheme of the theoretical modeling [47,52,53,54,55] is shown in Figure 2.

The thermal response on the laser irradiation can be found from the solution of the heat equation [47,53,55]

(1) $$c\rho \frac{\partial T}{\partial t}=div\;[k\cdot grad(T)]+U$$

Here, *c*, *ρ* and *k* are the values of specific heat, mass density and thermal conductivity, respectively; *T* is the temperature; *t* is the time. The solution of Equation (1) must satisfy the condition of continuity of temperature and normal component of heat flux at the boundaries of the contacting media.

A two-scale approach for calculation of kinetics of photo-induced temperature fields has been recently proposed [55,56]:

(i) *Macroscale model* is valid for mean temperature fields analysis in the spatially extended regions of tissues doped by assembles of plasmonic nanoparticles. In this case, the values *T*, *c*, *ρ*, *k* in Equation (1) should be considered as corresponding variables averaged over physically small volumes containing, at the same time, a sufficiently large number of nanoparticles.
(ii) *Microscale model* is valid for calculation of the small-scale spatial inhomogeneity of the temperature field within a nanoparticle itself and its vicinity. It means the exact local values of the variables *T* and other variables should be considered in Equation (1). This is important, e.g., for the study of cell membrane optoporation or transfection.

**Figure 3:**
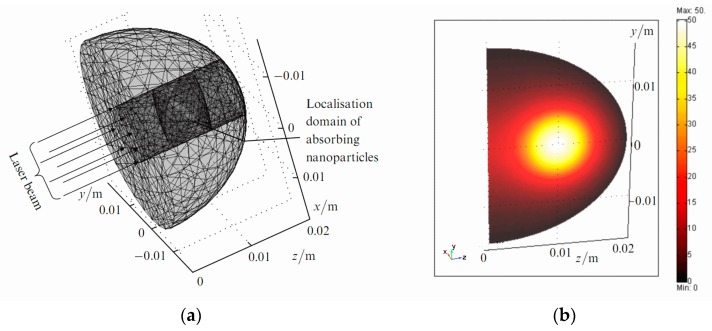
Three-dimensional finite-element grid in macro-domain (**a**) and distribution of temperature increment (°C) in the longitudinal sectional view of the macro-domain passing through the beam axis at the time moment of 300 s, corresponding to the end of light exposure (**b**). These figures are from [55] with permission of the Publisher.

The example of simulations for Model (i) is presented in Figure 3.

**Figure 4:**
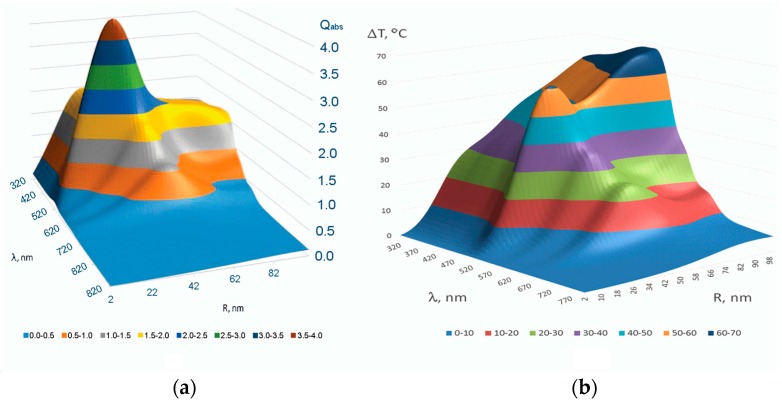
Distribution of the efficiency of absorption of the AuNP (**a**) [52] and of the temperature increment Δ*T* (**b**) on the surface of the AuNP versus wavelength *λ* and nanoparticle radius *R*; (**b**) is from [47] with permission of the Publisher.

Figure 4a represents [52] the distribution of the dimensionless value—efficiency of absorption $Q_{abs}=\int_{V}Udv/(\pi R^{2}I)$ versus radius *R* of Au spherical nanoparticle of the volume *V* = 4π*R*^3^/3 irradiated by laser light of wavelength *λ* and intensity *I*. The corresponding distribution of the steady state temperature increment Δ*T* within the Model (ii) for *I* = 100 kW/cm^2^ is depicted in Figure 4b.

**Figure 5:**
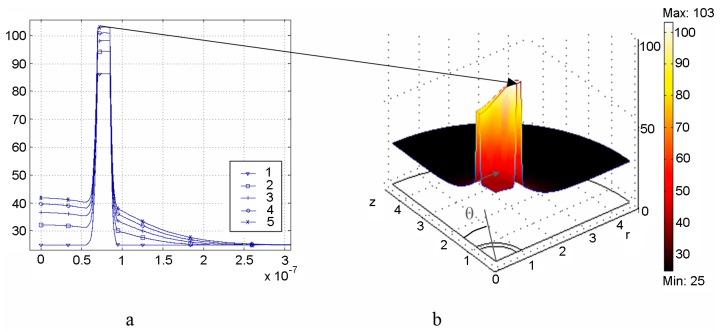
The temperature *T* distributions in the spherical nanoshell (with SiO_2_ core of a radius *R* = 70 nm and a gold coating of thickness 15 nm) in the water environment irradiated by a series of five pulses of a rectangular shape. The duration of each pulse is 50 ps, repetition rate is 10 ns, the intensity of radiation at a wavelength 800 nm is 4.5 MW/cm^2^: radial dependences of *T* at the times of turn-off of *j-*th irradiating pulse, *j* = 1/5 (**а**); two-dimensional distribution of *T* at the end of the 5th irradiating pulse (due to symmetry only 1/4 of the total allocation is presented) (**b**). The angle *θ* is measured from the axis *z*, directed along the polarization vector of the incident field. These Figures are from [53] with permission of the Publisher.

The short laser pulse model (ii) was developed as well. Under this approach, a novel thermal effect, which is a hoop-shaped hot zone formation on the surface of irradiated nanoshell, was found [53] (see Figure 5). The time of “life” of the hot zone is less than a nanosecond for the considered nanoshell size.

## 4. Arrhenius Damage Integral

The irreversible thermal damage of a particular type of a tissue or a cell is described by the condition for Arrhenius damage function or integral [46,47]:

(2) $$\Omega (r,\tau )=A\int_{0}^{\tau }\exp(-\frac{E_{a}}{R_{g}T(r,t)})dt\geq 1$$

Here, *τ* is the exposure time, *R_g_* = 8.314 J/(mol·K) is the gas constant.

For calculations, parameter *A* = 3.1 × 10^98^ s^−1^ and the activation energy *E_a_* = 6.3 × 10^5^ J/mol that characteristic for damage of porcine skin were used. Laser-irradiated AuNP creates a localized heated region in the surrounding medium. From Equation (2), the maximal allowed exposure time *τ* exceeding which leads to local destruction (damage) of cell membrane or tissue component can be determined [47].

**Figure 6:**
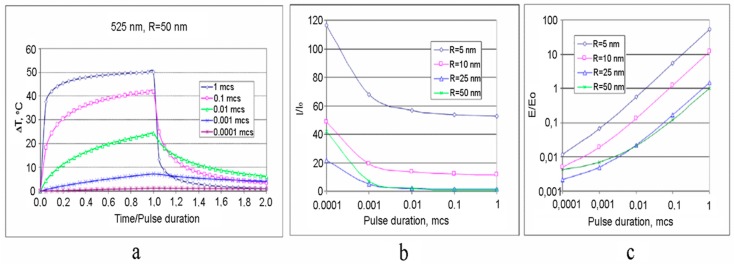
Temporal temperature evolution for 50 nm-AuNPs under the action of pulse laser radiation (*λ* = 525 nm) (**a**); The normalized power density (**b**) and pulse energy (**c**) of laser beam necessary for achieving the same temperature on the surface of AuNP, depending on pulse duration and nanoparticle radius *R* [52].

A study of impact of AuNP size and laser pulse duration on the temperature jump and damage function is of undoubted practical interest. Figure 6 shows temporal dependences of the temperature on the surface of a 50 nm-AuNP at different pulse duration *τ*_p_ which varies from 0.0001 to 1 µs [52]. The time *t* in Figuare 6a is normalized to pulse duration *τ*_p_. This allows for a graphical presentation in a single scale of relative time, time/pulse duration, and differently scaled temporal processes. The reduction of *τ*_p_ leads to a monotonic, however disproportional, decrease of maximal temperature jump Δ*T*_max_ at a similar laser intensity.

It is evident that the Arrhenius function Ω for various *τ*_p_ differs significantly due to two major factors, the maximal temperature rise Δ*T*_max_ and duration of the elevated temperature of the biological object, which both contribute simultaneously. It should be noted that the analysis would be more objective if the comparison were to be carried out for a similar Δ*T*_max_. A natural way to increase Δ*T*_max_ with a decrease of *τ*_p_ is the use of lasers with higher power densities. The required increase of the laser power to create an equivalent temperature effect can be estimated from data presented in Figure 6b. It is equally important to estimate the laser pulse energy *E*, which is required for the equivalent thermal exposure. Figure 6c, built in the coordinates “Normalized energy *E/E*_0_—Pulse duration *τ*_p_”, shows that larger AuNPs have the advantage of better energy efficiency compared to small-sized nanoparticles.

**Figure 7:**
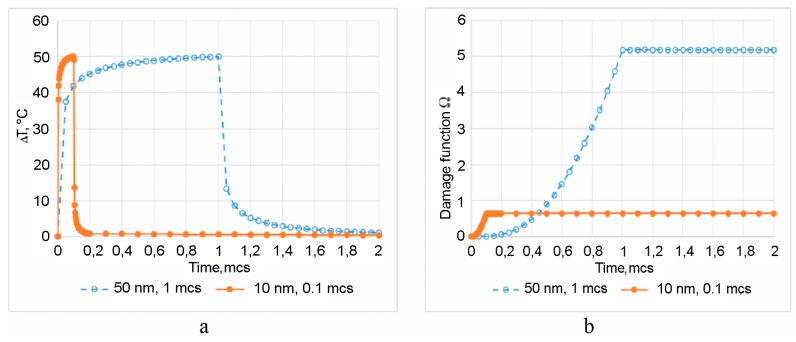
The temperature increments vs. time (**a**) and the Arrhenius integral Ω increments vs. time (**b**). Both curves correspond to the same laser pulse energy [52].

The results of calculations of Δ*T* and Ω for AuNPs (*R* = 50 nm, *τ*_p_ = 1 µs) and (*R* = 10 nm, *τ*_p_ = 0.1 µs) are shown in Figure 7 [52]. These AuNPs have a similar maximal pulse temperature received for the identical energy of the laser pulse. However, the increment of the Arrhenius function Ω, provided by AuNP with *R* = 50 nm and a longer pulse is almost an order of magnitude higher.

Results of this study show that the choice of NP size and laser pulse duration could provide a precise control of a local tissue/cell hyperthermia. At the same time, for both, the criteria for optimization are energy transform efficiency and economic feasibility. We see that the similar effects are achievable when using expensive femtosecond or relatively cheap nanosecond laser systems.

## 5. PPT/PDT Pathogen Killing Using AuNPs

The potential applications of AuNPs to kill pathogens, as well as to regulate the number of opportunistic microorganisms, are of great interest [57,58,59,60]. The use of AuNPs as light-activated agents, also in combination with other photosensitizers (PS), such as nanocomposites (NC) (see Section 2), increases the effectiveness of antimicrobial PDT.

The AuNPs-mediated PTT/PDT action of lasers working in the red and/or infrared spectral ranges is known to possess pronounced antimicrobial properties [61]. The main focus is on the suppression of growth of such clinically significant bacteria as *Staphylococcus aureus* (*S. aureus*) and *Pseudomonas aeruginos*a [16,62], which are characterized normally by a high resistivity to many present-day antibiotics. For targeted delivery of AuNPs, containing antibiotics or photosensitizers, such as chlorine e6 or toluidine blue, immunoglobulin molecules were successfully used [63,64].

In the study [65], use of gold(III) complex on nanoporous materials such as mobile composite material number 41 (MCM-41) at irradiation by 532 nm-laser light led to selective reduction of the number of fungi *Saccharomyces cerevisiae*. The AuNP-PS conjugates and 540 nm-irradiation allowed one to inactivate fungal biofilm of *genus Candida* [66,67]. A significant reduction of colonies *Candida albicans* and *Escherichia coli* (*E. coli*) was shown after their photoinactivation using AuNP complexes (nanorods and bipyramids) with aluminum phthalocyanine [66]. The AuNPs in combination with a polyurethane, phthalocyanine and methylene blue resulted in a significant decrease in the number of *S. aureus* [62,67,68]. At using a combination of gold nanorods with indocyanine green (ICG) photosensitizer (PS) and pulsed laser light (810 nm), a reduction in size of the *E. coli* bacteria was recently achieved [69].

In our studies [37,70,71,72,73,74], we used various modifications of AuNPs (nanorods, nanoshells, nanocages, nanoclusters) and their conjugates with a PS (ICG, HP, Photosens™ (PhS)) for the selective destruction of microorganisms by light exposure (see, Table 1).

Gold nanoparticles are unique objects for the targeted delivery of a variety of biologically active substances. Therefore, in the first phase of our research, the gold nanorods that conjugated with ICG were used for light-activated destruction of microbial cells [70]. We investigated the combination of NIR PTT/PDT against different bacterial strains (*S. aureus* and *S. epidermidis*, both methicillin susceptible and resistant). PTT was provided mostly at NIR light absorption by AuNPs, as PDT, by ICG molecules. The absorption spectrum of ICG bound to cell structures overlaps well with the plasmonic resonance of the gold nanorods (~800 nm). Therefore, diode laser 808 nm-radiation with a power density of 50 mW/cm^2^ inhibited growth of *S. aureus* by 65% and *S. epidermidis* by 80% via combined PPT/PDT treatment with a very low overheating of the cell suspension, which did not exceed 6 °C.

Other types of gold nanoparticles conjugated with ICG molecules were also used; for instance, silica/gold nanoshells covered by additional mesoporous silica shell (about 10–15 nm) that was functionalized with ICG molecules [70,71]. The IGG molecules were absorbed on the particle surface due to the electrostatic interaction with positively charged amine groups. Additionally, the use of gold-silver nanocages with an inner cavity for containment of ICG molecules was described in [70]. Laser radiation of 805 nm (46 mW/cm^2^) for nanoshells and 808 nm (60 mW/cm^2^) for nanocages demonstrated a similar inhibitory effect for *S. aureus*.

It should be noted that the concentrations of ICG used in some studies in combination with NIR laser radiation (810 nm, 48 mW/cm^2^, 30 min) were relatively high (25–250 mg/mL) [75]. Under these conditions, the suppression of bacterium growth by 90%–99% was observed for *S aureus*, *P. aeruginosa*, and *Streptococcus pyogenes*. In our experiments, the dye concentration was 1–2 orders of magnitude lower (2.5 mg/mL). Even at such low concentration of ICG, we observed a pronounce suppression (by 75%) of the meticilline-sensitive *S. aureus* 209 P.

In search of new effective and low-toxic conjugates of nanoparticles and PDT dyes, we have chosen further studies of gold nanorods and gold-silver nanocages in complex with hematoporphyrin (see Figure 1) [37]. The effective suppression of *S. aureus* by dual PPT and PDT action was studied and compared with the activity of the control solutions (1—hematoporphyrin, 2—plasmonic nanoparticles coated by silica with concentrations similar to use in conjugates).

To irradiate nanocomposites, we used a red LED (625 nm, 33 mW/cm^2^) and a NIR laser (808 nm, 100 mW/cm^2^), respectively. At irradiation with 625-nm light, the nanoconjugates have an enhanced PDT action toward *S. aureus* bacteria (80%–97% cell death), as compared with a molecular solution of HP of the similar concentration. An additional photoinactivation of bacteria can be provided by PPT action under irradiation of nanoconjugates with NIR light at a wavelength close to the plasmon resonance. Incubation of the bacteria with nanoconjugates and their irradiation at 808 nm also lead to decrease of bacterial survivability (65%–90% cell death). Because the mean overall heating of the cell suspensions was insignificant in this case, a possible mechanism responsible for the injury to the bacteria can be the local heating of nanoconjugates at the cell wall because of light absorption at the wavelength of the plasmon resonance of the composite’s core.

**Figure 8:**
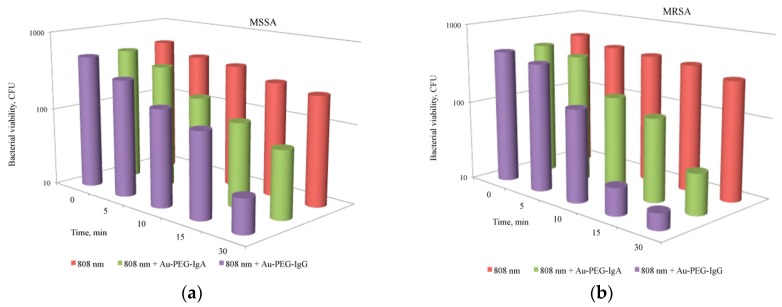
Action of NIR laser light (808 nm) and gold nanorods conjugates with immunoglobulin A and G on *S. aureus* survival rate: methicillin sensitive strain (MSSA) (**a**); methicillin-resistant strain (MRSA) (**b**). Red columns—laser light, green columns—laser light and AuNR3 with IgA, violet columns—laser light and AuNR3 with IgG.

The improvement of efficiency and selectivity of the photothermal effect of laser (808 nm) radiation against both antibiotic-sensitive and antibiotic-resistant strains of *S. aureus* is possible by using novel modifications of the “antibody–nanoparticle” system. In our studies, we offered a system of gold nanorods that carry on their surface inverted Fc-fragments of human immunoglobulin A and G [71,73]. The Fc-fragments of IgG in conjunction with nanoparticles have demonstrated a greater effectiveness than IgA (Figure 8). The number of MSSA cells in suspension with gold nanorods decreased by 78% (CFU reduction) after 15 min of NIR radiation exposure, and by 95% after 30 min exposure. For the MRSA strain, the number of cells incubated with IgG-conjugates of gold nanorods decreased by 96% after 15 min of light exposure and by 97% after 30 min.

The increase of mean temperature in the bacterial suspension depended on the light exposure, the maximal values being recorded after 30 min of NIR irradiation. In the experiments on the impact of NIR radiation in combination with gold nanorods, the number of microorganisms of two studied strains decreased proportionally to the increase in the mean temperature. This means that the major contribution to the damage of bacterial cells can be related to the local photothermal plasmon resonance heating rather than to the total heating of suspension.

Under laser irradiation, the increase of mean temperature of suspensions of 12.7 °C for nanoparticles conjugated with IgA and of 15.2 °C for nanoparticles conjugated with IgG was found, as compared to heating of a pure NP suspension. This phenomenon can be associated with the better stability of the colloidal system due to formation of clusters of nanoparticles and bacterial cell wall via immunoglobulin Fc-fragments, which may lead not only to better efficiency of the local photothermal effects, but also to elevation of suspension mean temperature.

Functionalization of nanoparticles by antibodies allows one to use a wide range of auxiliary components and various synthesis conditions. Recently, we described Au-BSA nanoclusters [74] functionalized with targeting molecules (human anti-staphylococcal immunoglobulin, IgG) and PDT dye Photosens™ (PS) [76] for selective detection and effective PDT inactivation of both methicillin sensitive and resistant *S. aureus*.

**Figure 9:**
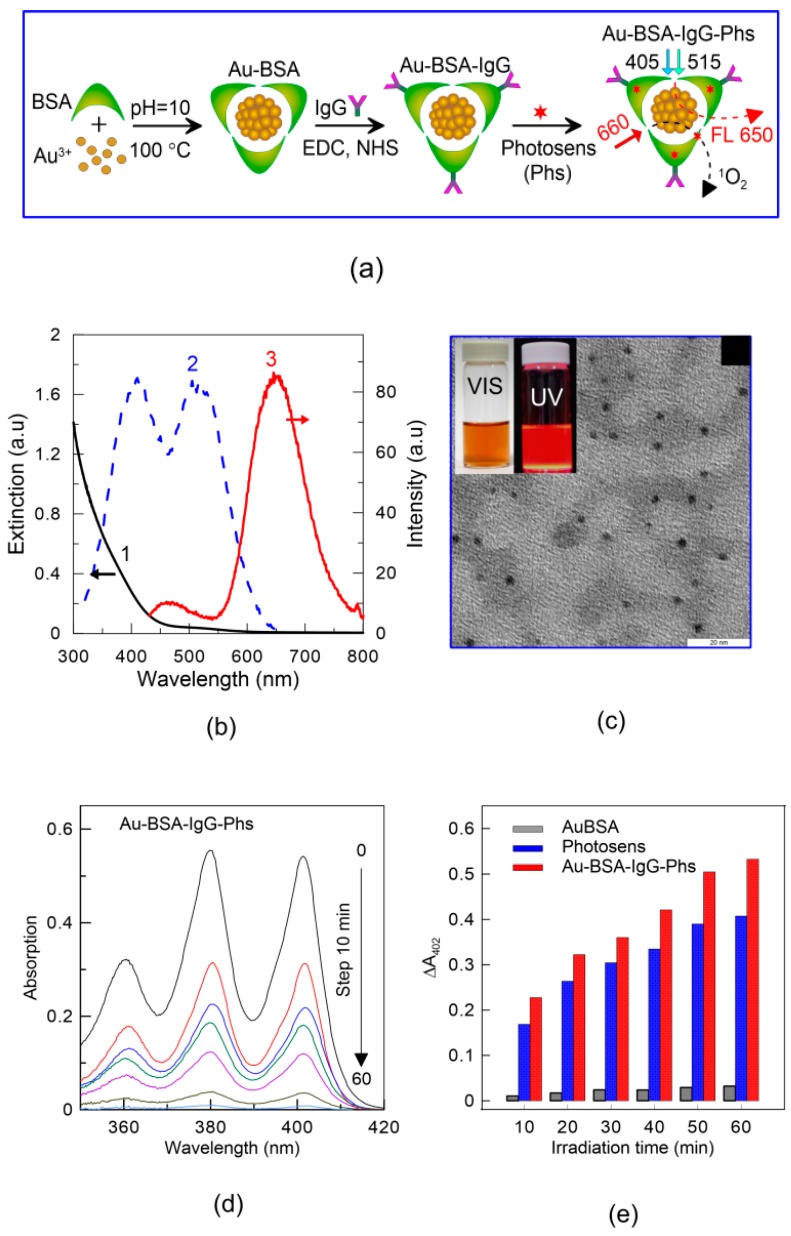
Scheme of the preparation of the Au-BSA-IgG-PS complexes and their fluorescent and PDT properties under 405, 515 nm and 660 nm-excitation, respectively (**a**); extinction (1), excitation (2), and emission (3) spectra of Au-BSA NCs (**b**); TEM images of Au-BSA NCs, the insets show the photos of the solutions under white light (**left**) and UV light (**right**) irradiation, The scale bar is 20 nm (**c**); absorption spectra of a mixture of ABDA and Au-BSA-IgG-Phs under illumination by a 633-nm laser for different exposures from 0 to 60 min (**d**); the absolute changes in ABDA absorbance at 402 nm and as a function of irradiation time; the gray, blue, and red columns correspond to incubation of bacteria in Au-BSA NCs, Photosens solution, and Au-BSA-IgG-PhS NCs, respectively (**e**). Reproduced from [74] with permission from The Royal Society of Chemistry.

The synthesis of Au-BSA-IgG-PS complexes included three consecutive steps shown schematically in Figure 9a. Initially, Au-BSA nanoclusters were prepared from a mixture of BSA and Au^3+^ at high pH and boiling temperature [77]. The main characteristics of received Au-BSA nanoclusters are given in Figure 9b—extinction (1), excitation (2) and emission spectra (3). In general, the extinction spectrum is similar to the spectrum of small AuNPs (<3 nm), where the plasmonic peak around 510–520 nm is also missing. The large or aggregated nanoparticles were absent as demonstrated in TEM images of nanoclusters (Figure 9c), and the average size of Au-BSA nanoclusters is about 2 nm. In aqueous solution, the as-prepared Au-BSA are highly dispersed and they exhibit strong red fluorescence (FL) under UV illumination and brown color under white light, as shown in the insert of Figure 9c. The curve 2 in Figure 9d demonstrates two peaks around 405 and 514 nm in fluorescence excitation spectrum of Au-BSA nanoclusters. The emission maxima are found near 470 and 660 nm. As defined by using hematoporphyrin as a benchmark standard, the quantum yield of Au-BSA nanoclusters was about 14% at optimum conditions. The application of BSA as a coating agent is preferred because it contains various functional groups which could be used to bind with different ligands. Furthermore, the high sorption capacity of BSA for PS dyes, drugs and various therapeutic agents enables the use of BSA-capped nanoclusters as a promising nanoplatform for theranostic purposes. One of the possible applications has been demonstrated in Step 2, in which Au-BSA nanoclusters were functionalized with human anti-staphylococcal IgG, which are also known as antibodies against *S. aureus* endotoxin. In the last stage, Au-BSA and Au-BSA-IgG complexes were conjugated with photosensitizer (PS). Due to the high affinity of PS to BSA and other serum proteins, PS molecules were successfully incorporated into the BSA matrix and multifunctional Au-BSA-IgG-PS complexes were formed.

The photo-oxidation of ABDA in the presence of AuNCls and Au-BSA-IgG-PS complexes was measured under LED laser irradiation (660 nm) for 60 min. As shown in Figure 9e, the characteristic absorbance maxima of ABDA, in particular the main peak at 402 nm, gradually decreased with an increase in light exposure. A weak photooxidation ability of Au-BSA nanoclusters itself was also revealed (data not shown here). The synthesized Au-BSA-IgG-PS complexes show enhanced PDT activity that is comparable or even a bit higher than that for free PS solution.

Thus, such Au–BSA–anti-SAIgG complexes, due to their biospecific targeting and intense red fluorescence, may identify pathogenic microorganisms in bacterial suspensions by FL microscopy or even by the naked eye in the investigation of sediments under UV illumination. The proposed nanocomposites may be applied at a physiological pH of 7, as opposed to nonspecific electrostatic binding of Au—human serum albumin AuNCls to *S. aureus* at pH < 5–6.

It was shown that exposure to red (660 nm) radiation and Au–BSA–anti-SAIgG complexes leads to clearly marked destruction of two studied Staphylococcus strains (MSSA and MRSA). If the red (660 nm) light in the absence of photoactive agents ensured destruction of microorganisms at a rate of 75% and 63%, respectively, then the adding of total Au–BSA–anti-SAIgG complexes to the suspension resulted in the death of up to 90% of bacterial cells.

Protein- or glutathione-coated AuNCls appear to be a more convenient platform for nanomedicines than commonly used large AuNPs. Really, the presence of stable fluorescence in AuNCls makes them an indispensable tool for nanodiagnostics, without the need to functionalize them with FL dyes as well. These NCs were used as sensitive markers for the detection of pathogenic bacteria; for example, selective sensitivity was found against MSSA and MRSA in [74], where we established pH-dependent selective binding of Au—HSA NCs to MSSA and MRSA.

Thus, the gold nanoparticles in various modifications significantly increase the effectiveness of PDT treatment of bacterial infections. Various mechanisms are apparently involved in killing of bacteria, including the local increase in the concentration of the photosensitizer through targeted delivery of nanoparticles, selective interaction with the cell wall of bacteria, and the resonance heating of AuNPs under laser light irradiation.

## 6. Photothermal and Photodynamic Therapy for Transplanted Tumors

To demonstrate the utility of fabricated Au nanorods and Au nanocomposites for in vivo applications, we investigated the PTT and PDT treatments for big solid tumors in rats. 

Recently, several research groups reported the use of various gold nanoparticles: nanoshells, nanorods, nanocages, and other nanocubes for the plasmon resonance hyperthermia [23,78,79,80,81,82]. Use of gold nanorods (AuNRs) for PTT is preferred due to the colloidal stability and easy customization of nanorods plasmon resonance in accordance with the laser wavelength by changing the axial ratio of nanoparticles [83]. To improve the biocompatibility of the nanoparticles and to enhance their stability, different biocompatible polymers are applied [84]. A longer circulation time and better accumulation in tumors show nanoparticles coated with neutrally charged polymers, including polyethylene glycol [85]. We have previously tested a method of photothermal plasmon-resonance therapy in tumor-bearing rats with alveolar liver cancer PC-1 rats with the intratumoral introduction at an amount of 30% of the tumor volume was intratumorally injected. The length of AuNRs was 41 nm ± 8, diameter was 10 ± 2 nm, concentration was 400 µg/mL, and a maximum absorption was noted at a wavelength of 808 nm corresponding to the plasmon resonance of the gold nanorods. One hour after, laser irradiation was carried out percutaneously over the surface of the tumor for 15 min. The 808-nm CW dide laser LS-2-N-808-10000 (Laser Systems, Ltd., St. Petersburg, Russia) with a power density 2.3 W/cm^2^ was used for the laser hyperthermia. Temperature control of tumor heating was performed every 30 s using infrared thermograph IRI4010 (IRYSYS, Northampton, UK). During the laser irradiation a significant rise in temperature (55 ± 2 °C) was noted, most pronounced in the first 2 min of irradiation. Twenty-four hours after laser hyperthermia, animals were removed from the experiment, and the marked changes were revealed at morphological study of tumors. The necrotic zones occupy 80%–90% of the slice area in tumors. Nevertheless, survived tumor cells with degenerative changes were detected only in the subcapsular area of the tumors.

The aim of the next study was to investigate the combined PDT and PTT treatment of tumor-bearing rats [38]. We were interested in the impact of large tumors, therefore, white outbred male rats with implanted cholangiocarcinoma PC-1 were taken in experiments on reaching their tumor volume of about 3 cm^3^. Five animal groups were formed randomly (6 rats per group): the control group received only saline injection (group I), comparison group received saline injection and was treated with laser 808 nm irradiation (group II), group III received NC injection and was treated with laser 808 nm irradiation (PTT), group IV received NC injection and was treated with laser 633 nm irradiation (PDT), group V received NC injection and was treated with synchronous irradiation of laser 808 nm and laser 633 nm (PDT + PTT). All injections were made intratumorally. The power density of 808-nm CW diode laser LS-2-N-808-10000 (Laser Systems, Ltd.) was 2.3 W/cm^2^ (groups II, III and V), the power density of 633-nm CW laser (GN-5P, “Plasma” Corp., Ryazan, Russia) was 160 mW/cm^2^ (group IV and V). Finally, animals of group V were simultaneously irradiated with both lasers. Each irradiation treatment continued for 20 min. The surface temperature profile over the tumor was captured using infrared camera IR Imager IRI4010, Infrared Integrated System (IRISYS). The tumor biopsies were sampled three days after the laser exposure, hematoxylin and eosin (H & E)-staining was used for morphological examination.

In comparison group II, only NIR irradiation caused a slight increase in the surface tumor temperature from 30 °C to about 40 °C, thereby, necrotic changes were not observed in tumor tissues. Nevertheless, at 808-nm laser irradiation in rats with NC-inection, the ablative values of tumor temperature rapidly exceeded 60 °C and were maintained at about 75 °C thereafter. It is known that rapid coagulative necrosis and irreversible cell and tissue damage were observed at temperatures above 70 °C.

The simultaneous treatment of tumors with NIR and He–Ne lasers also results in an increase of surface tumor temperature, which was slightly higher compared to PTT group. Therefore, PDT contribution to the total thermal effects was insignificant in group V. Furthermore, the temperature of the NC-treated tumors did not increase at only 633-nm irradiation, indicating that no thermal effects were induced under PDT.

**Figure 10:**
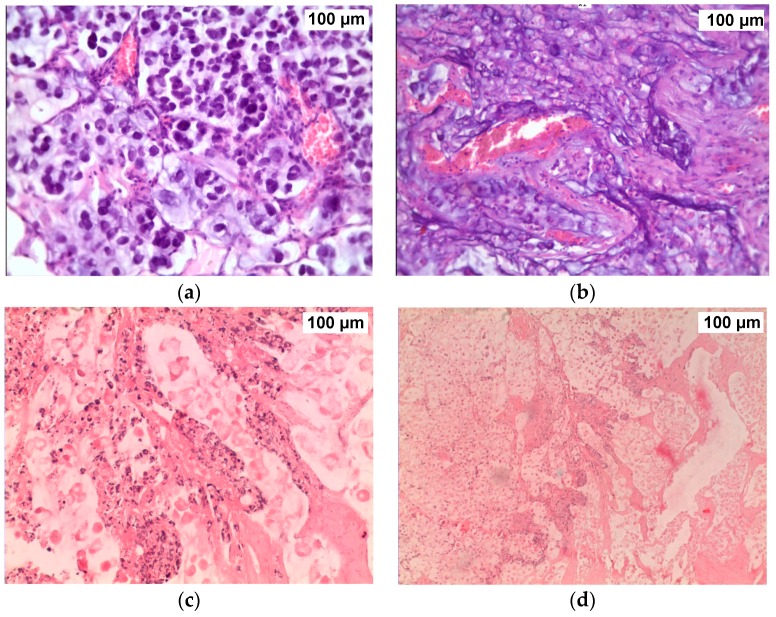
Liver tumor slices after different treatments: Comparison group with 808 nm laser irradiation only (**a**); PDT-group (**b**); PTT group (**c**); PDT + PTT group (**d**). H & E staining, ×246.4. Reproduced from [38] with permission from the Springer and Wiley.

Morphological investigations of treated tumor tissues revealed the negligible effect of only NIR laser irradiation on cancer cells in the comparison group (Figure 10a). In group III (NCs injected + 633 nm laser irradiation), an increased number of brown spots were noted in tumor tissue, indicating some apoptotic damage of cells after PDT treatment (Figure 10b). In PTT-treated group (NCs injection + NIR laser irradiation), large areas of necrosis appeared in tumor tissue after NIR-irradiation (Figure 10c). Finally, in group V, marked necrotic changes were revealed in tumor tissue and significant tissue loss was observed after combined PDT + PTT treatments (Figure 10d).

Simulated and experimental studies demonstrated that photothermal heating effects could be quite complicated and depend on the nanoparticle design and irradiation conditions [21]. The nanoparticle characteristics such as size, plasmonic resonance value, the type of the surface coating, and the particle concentration affect increase of tumor temperature and thus determine photothermal treatment efficiency. In addition, the heating also depends on irradiation parameters including laser wavelength, laser power and treatment time. To achieve appropriate temperature increments in xenografted tumors in vivo, the laser power density in the range of 1–5 W/cm^2^ is used and the treatment exposure varies between 1 and 10 min [80,82,83].

A few previously performed in vivo studies of combined PDT/PTT therapy with using different types of GNPs focused on the treatment of small tumors (typically, less than 0.5 cm^3^) [80,82,83]. The effective therapy of larger tumors poses new challenges related to the route of nanoparticle administration and their tumor accumulation, deep penetration of laser radiation inside the tumor, and optimization of NC and irradiation doses [86]. The NIR laser power density of 2.3 W/cm^2^ applied during 15–20 min has been shown to be effective for PTT damage of large tumors at intratumoral injection of gold nanorods [38,87].

Recently, an interesting solution was proposed by Boseung Jang et al. [19] for PTT/PDT treatments to increase the damage to SCC7 squamous cell carcinoma tumors in Balb/c-nu mice in vivo*.* The nanocomposites based on 34 nm AuGNRs (aspect ratio (AR) 3.7, λ_max_ ~800 nm) and functionalized by RRLAC peptide were further conjugated with a photosensitizer AlPcS4 (absorbance at 675 nm) via electrostatic immobilization. The photothermal heating of tumors caused the release of bound photosensitizers from nanocomposites. PTT (810 nm, 3.82 W/cm^2^) followed up by PDT treatment (670 nm, 331 mW/cm^2^) which caused an increase of tumor temperature up to 65 °C.

The lower laser power density is usually applied for PDT, because the main effects of photodynamic treatment associated with singlet oxygen generation but not with tissue hyperthermia.

In our work [38], we have showed effective combined PDT/PTT treatment for large solid tumors, whereas only PDT treatment was ineffective for antitumor therapy. Nevertheless, possible tumor recurrence, probably caused by the limited light penetration and none-optimal spatial distribution of the NIR laser radiation within the tumor, remains an unsolved problem. Thus, further studies are needed to improve the therapeutic protocols by correct selection of nanoparticle administration techniques and irradiation modes for deep light penetration and adequate damage to tumor tissue.

## 7. AuNP Mediated Optoporation

Recently, a new AuNPs mediated technique for permeabilizing cells was introduced [88]. In this method, AuNPs were placed on cells and treated by a weakly focused laser beam, which leads to a significant increase of membrane porosity in the vicinity of AuNPs. Spectral localization of LSPR enormously enhances laser absorption leading to photothermal and related phenomena such as heating of the surrounding medium/tissue, microbubble formation and acoustic shockwave generation [89,90]. Notably, that for treatment efficiency and locality, the laser treatment of cells should be optimized. From this point of view, it is crucial to distinguish the difference between impact on the cell/tissue caused by an enhanced localized thermal laser action mediated by AuNPs and non-targeted laser irradiation of the surroundings [91,92].

The mechanism of gold nanoparticle mediated (GNOME) optoporation and transfection is based on laser perforation caused by short laser pulses of sufficiently high intensity, which leads to formation of vapor nanobubbles (NBs) [93,94,95]. One of the first applications of laser-induced transient vapor NBs around overheated gold nanoparticles (called also photothermal or plasmonic NBs) in biology and medicine with a focus on integration of cancer and pathogen detection and treatment was performed by Zharov’s group in 2003 [90]. NBs emerge due to a rapid increase of the AuNPs’ temperature to several hundred degrees and evaporation of water surrounding AuNPs [96,97]. Collapse of NBs causes local damage to cell membranes. For successful delivery of medical drugs, DNA, or RNA molecules into the cytoplasm, cell membranes can be perforated, without any permanent membrane damage [98]. Due to the extremely short lifetime of NBs, the diffusion of heat from the AuNPs into the surrounding medium is negligible.

The applicability of GNOME laser optoporation and transfection for cell manipulation was intensively studied during the last decade [88]. Typically, to mark perforated cell membranes under laser treatment, one adds fluorescent dyes, which are unable to introduce untreated cell membrane, in cell suspension [99]. Pitsillides et al. in 2003 were among the first to demonstrate the increased permeability of cell membrane in the presence of AuNSps under 10-kDa fluorescein–dextran conjugate in the presence of AuNSps under 20-ns, 532-nm laser pulses with an energy density of 0.5 J/cm^2^ [100]. The biological consequences of GNOME were investigated in detail by Heinemann’s research group, with analysis of the potential changes to the cell membrane [88]; study of cell volume and area and ion exchange [101]; calculation of the kinetics of fluorescent dyes perforation [102]; and evaluation of perforation process of fluorescent dextrans in a size range of 10–2000 kDa under irradiation of a 532 nm picosecond laser as a function of irradiation time and repetition steps [103]. For all research works demonstrated below, AuNSps were attached to the cell membrane due to a sedimentation process. To achieve selective targeting of cell membranes by nanoparticles, functionalization by specific antibodies is needed. Addressing this, Cuiping Yao et al. functionalized 15 nm and 30 nm AuNSps by antibodies to the plasma membrane of the Hodgkin’s disease cell line and/or the human large-cell anaplastic lymphoma cell line [104,105]. Strong absorption ability allowed AuNSps with LSPR at 532 nm to perforate the cell membrane even when the irradiation wavelength of the nanopulsed laser is off resonance at NIR (1064 nm), which maximizes the penetration depth and opens up the possibility to reach sublayer cells in vivo [106,107]. Notably, the AuNPs’ morphology and colocalization with cells (either target the cellular membrane or are endocytosed) should also be taken into account. AuNRs and hollow AuNSps induce less cell damage than AuNSs under picosecond irradiation [108]. Anisotropic AuNP polarization should be taken into account as well. Recently, the importance of AuNRs’ orientation to the incident electric field was acknowledged, where local defects in the phospholipid cell membrane were only induced when nanorods were placed in parallel to the polarization of the electric field of the laser beam, a normal orientation of AuNRs to the polarization field [109]. The energy density of the laser pulse that is necessary for effective cell perforation strongly depends on the molecular weight of the delivered material [4]. However, in spite of the differences between laser sources used by different research groups, energy densities providing cell optoporation are in the range of 100–200 mJ/cm^2^ with the threshold value of 15–30 mJ/cm^2^ [99,100,101,102,103,104,105,106].

Recently, we demonstrated different optoporation abilities in a comparative study of AuNPs with variable morphology for evaluation of their impact on cell membrane permeability under irradiation by three laser sources operating at different modes and wavelengths [110,111]. These lasers were used to irradiate the HeLa cells: CW-laser (Aculaser, Inc., Las Vegas, NV, USA), with a central wavelength 808 nm, power 2.5 W, and beam diameter 5 mm; a ns-laser (Opotek Tunable Laser Systems, OPOTEK, Inc., Carlsbad, CA, USA) generated laser pulses at 532 nm with a pulse duration of 5 ns, repetition rate 20 Hz, maximal pulse energy 0.1 mJ, mean power 2 mW, and beam diameter 5 mm; a nanosecond ytterbium fiber laser (scan-ns-laser) (Mini Marker 2TM, Laser Center, St. Petersburg, Russia) in 3D scanning mode, with the wavelength 1064 nm, pulse duration of 4 ns, repetition rate up to 20 kHz, pulse energy up to 1 mJ, mean power 20 W and sharply focused beam with diameter 6 µm. For 3-D scanning process, a laser beam scanned the sample surface area of 3 × 3 mm^2^ in the horizontal XY plane with the step of 20 μm. Then, the focal point was stepped down along *Z*-axis with the step of 25 μm to provide a three-dimensional irradiation matrix. The total number of laser pulses on one focal plane was 4 × 10^4^ with the scanning time of 2 s, and the scanning speed of 0.4 m/s, i.e., one pulse per irradiation point. The scanned depth along *Z*-axis was 2 mm. Single pulse energy was taken as 0.1 µJ. For the experiments, we used three types of AuNPs: nanostars (AuNSts) with LSPR 805 nm [112], AuNSps with LSPR 520 nm [113], AuNRs with PR 805 nm [114]. The biocompatibility of synthesized AuNPs was discussed elsewhere [115,116], the chosen concentration of AuNPs 17 μg/mL was sufficient for cell perforation and was harmless to living cells according to previous reports [107]. Investigation of optoporation kinetics was performed of the fluorescent dye propidium iodide (PI), which becomes fluorescently detectable as a result of binding to nucleic acids after membrane permeabilization [117]. The exposure time to PI on the cells was essentially shorter in comparison with the classical staining protocol [118], to find only perforated cells as PI-positive. A similar method was applied in Meunier’s research group [107], as well as Kalies et al.’s [102] who studied the efficacy of Lucifer Yellow dye uptake depending on laser exposure to evaluate membrane permeabilization.

MTT test was performed under irradiation conditions similar to the PI test to determine cell viability. To evaluate the heating properties of the AuNPs surrounding media, temperature measurements were carried out every 15 s during the laser irradiation.

**Figure 11:**
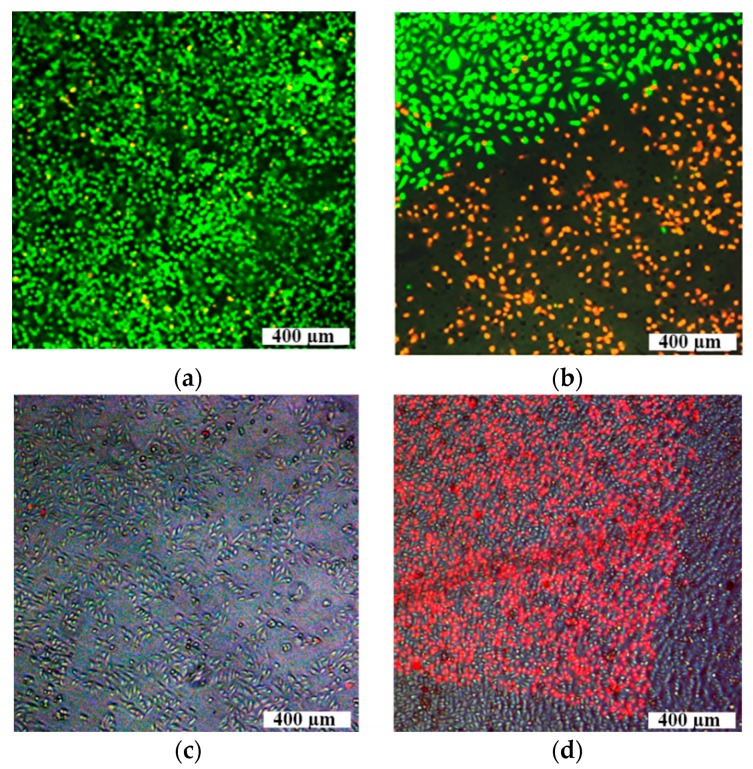
**Upper** row: the fluorescent images of cells stained by Calcein AM (green color) and PI (red color) irradiated by ns-laser generated laser pulses at 532 nm with a pulse duration of 5 ns, repetition rate 20 Hz, maximal pulse energy 0.1 mJ, mean power 2 mW: samples of pure cells (**a**); cells incubated with AuNSps under NS-laser treatment (**b**); **Lower** row: combination of bright-field and fluorescent images of cell samples irradiated by the scan-NS-laser with a wavelength of 1064 nm, pulse duration of 4 ns, repetition rate up to 20 kHz, mean power 20 W, and single pulse energy of 0.1 µJ: without AuNSts (**c**); with AuNSts (**d**). PI-perforated cells stained red color.

After irradiation by CW laser, samples of cells incubated with AuNRs demonstrated the highest percentage of PI-positive cells under laser treatment, thereat MTT assay showed almost total cell death as a result of strong heating of the surrounding medium in the presence of AuNPs (increase up to 72 °C after 2-min irradiation) leading to cell damage and death [110]. In contrast, we have not observed any temperature increase of cell medium in the presence of AuNPs under ns-laser irradiation. Figure 11 demonstrates the fluorescent images of cells irradiated by ns-laser generated laser pulses at 532 nm with a pulse duration of 5 ns, repetition rate of 20 Hz, maximal pulse energy of 0.1 mJ, mean power of 2 mW without AuNSps (a) and with AuNSps (b); and combination of bright-field and fluorescent images of cell samples irradiated by the scan-ns-laser with a wavelength 1064 nm, pulse duration of 4 ns, repetition rate up to 20 kHz, mean power of 20 W, and single pulse energy of 0.1 µJ: without AuNSts (c); with AuNSts (d). In the upper row, fluorescent dye Calcein AM stained only undamaged cells.

**Figure 12:**
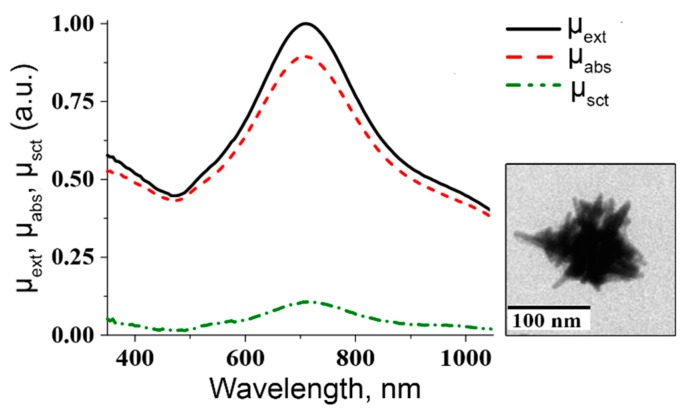
Typical spectra of AuNSts extinction (μ_ext_), absorption (μ_abs_), and scattering (μ_sct_), coefficients and representative TEM image of single AuNSt (**inner part**).

For both types of nanosecond lasers, the border of the collimated laser beam is clearly seen for cell suspensions incubated with AuNPs: inside the irradiated area, only perforated cells are present. Effectiveness of AuNSts mediated optoporation with nanosecond laser in 3D scanning mode resulted from the broad plasmonic resonance of AuNSts, where, at a laser irradiation wavelength of 1064 nm, absorption is still high for successful optoporation, and a relatively high contribution of absorption to extinct parts of the AuNsts spectrum can be made. Figure 12 shows typical spectra of AuNSts’ extinction (μ_ext_), absorption (μ_abs_), and scattering (μ_sct_), coefficients, the peaks of which can vary from 650 to 1000 nm dependent on nanoparticle size.

Recently the mutual dependence between the diameter of NSts and absorption-scattering cross-section ratios (C_abs_/C_sca_) was shown experimentally. For the relatively small NSts (50 nm diameter), the contribution of scattering to absorption was much smaller, than for large NSts (diameter more than 100 nm) [119], which corresponds to the theoretical investigation undertaken by Yan et al. for nanostars and Prashant Jain et al. for AuNPs with variable morphology [120]. Therefore, to maximize the potential influence of laser irradiation on the AuNPs, it is necessary to fabricate AuNPs with high absorbance and LSPR corresponding to the wavelength of the laser source. Effective cell permeabilization with a precise control of cell treatment allows applying AuNPs’ optical transfection: an effective and accurate method with many potential applications both in vivo and in the clinic. The ability to deliver DNA/RNA/siRNA into mammalian cells using AuNSps and AuNRs was demonstrated by Ripken’s [121,122], Chia-Chun Chen et al.’s [123] and Dholakia’s research groups [124]. Even more precise treatment can be achieved by single-cell transfection techniques [125,126], which enable the individual monitoring and performing of genetic changes in a specific cell, without treatment of other cells.

## 8. Conclusions

The therapeutic efficacy of only PDT in cancer treatment is limited because of the inadequate selectivity of most photosensitizers and their poor solubility and rapid destruction under light exposure. A combination of photodynamic therapy (PDT) and photothermal therapy (PTT) may become a universal and gentle tool for biomedical applications in the near future, which allows one to avoid side effects and the development of resistance to drugs. Development of AuNP-based nanocomposites combined with PDT agents can improve the effectiveness of both PDT and combined therapies.

Currently, integrative approaches are required for precise control of laser thermal action of PTT with nanoscale/femtosecond resolution, which combined existing cutting-edge and newly developed methods for mathematical modeling of the optical and thermal properties of nanoparticle-containing medium. A general conclusion can be made about the significance of size-dependent distribution of AuNPs to provide optimal local laser hyperthermia of cells and tissues. The use in numerical simulations of the Arrhenius damage integral allowed us to make a more reasonable choice of the nanoparticle size and irradiation mode to precisely control hyperthermia with minimal energy consumption.

The proposed approaches can be used for prognosis and monitoring of local hyperthermia caused by application of nanophotosensitizers of different shapes/structures and laser irradiation. 

Furthermore, the considered theoretical and experimental reports can be useful for a better understanding of new applications of AuNPs to pathogen killing or PTT/PDT treatment of tumors. Further studies are needed to develop a robust and effective strategy for delivery of AuNPs and related composites to tumor tissue, thus ensuring an effective dual PTT/PDT effect.

We have evaluated the cell membrane optoporation percentage in in vitro experiments in terms of fluorescent dye permeability under CW and nanosecond (ns) pulse laser treatments. Differently-shaped AuNPs as nanospheres, nanorods and nanostars with various plasmon-resonant peaks were fabricated and functionalized with different ligands to achieve the optimal AuNP–cell interaction. More than 85% of cells can be permeabilized within the illuminated area. Nanostars demonstrated the highest optoporation efficacy under pulse laser irradiation at 1064 nm. The optoporation technology based on AuNPs with high absorbance seems to be an effective method for cell permeabilization with a precise control of cell treatment. This makes this novel technique a prospective tool for highly efficient transfection of extracellular substances into cells.

## Figures and Tables

**Table 1:** Nanoparticles, PSs and experimental conditions for treatment of pathogens.

Abbr.	Nanoparticle Shape	Photosensitizer (PS)	Functional Component	Average Size, nm	Type of Radiation	Maximal Inhibition of *S. aureus* 209 P after 30 Min-Light Exposure; CFU, % (Reference)
AuNRd1	Nanorods	ICG	–	30 × 10	808 nm, 50 mW/cm^2^	65 [70]
AuNS	Nanoshells	ICG	–	140	805 nm, 46 mW/cm^2^	55 [71]
AuNCg	Nanocages	ICG	–	53	808 nm, 60 mW/cm^2^	64 [71]
AuNR2	Nanorods	HP	–	50 × 10	808 nm, 100 mW/cm^2^	90 [37]
AuNCg2	Nanocages	HP	–	50	625 nm, 100 mW/cm^2^	97 [37]
AuNR3	Nanorods	–	FcIgA, FcIgG	45 × 13	808 nm, 100 mW/cm^2^	95 [72,73]
AuNCl	Nanoclusters	PhS	BSA + IgG	1.8 (25 Au atoms)	660 nm, 50 mW/cm^2^	90 [74]
